# Book Reviews

**Published:** 1862-04

**Authors:** 


### OUR LIBRARY TABLE.

*On the Influence of Tropical Climates in producing the Acute Endemic Diseases of Europeans; including Practical Observations on the Nature and Treatment of their Chronic Sequelæ, under the Influence of the Climate of Europe.* By SIR JAMES RANALD MARTIN, C.B., F.R.S., Physician to the Council of India. 2nd Edition. 8vo. London, 1861. Pp. 778.

It would be idle and somewhat impertinent to subject to any detailed criticism a work which has on all hands, and most justly, been received as the standard authority in the English language on the subjects of which it treats. This book is simply a necessity for every medical man who may be called upon to practise in tropical climates, or to treat the chronic sequelæ of tropical diseases at home. Nor is its importance restricted within these limits, for it is equally a necessity for every practitioner whose idea of the science of medicine is not bounded by notions of latitude and longitude. The present edition of Sir James Ranald Martin's work is not, it is necessary to note, a mere reprint of the former edition. He has added much, altered some things, and re-arranged his materials; in fact, he has elaborated the work, and so far augmented its value.

The whole subject of the influence of tropical climates upon Europeans will become of increased interest to the profession and the public when the Annual Statistical Report of the Army shall include the returns from the force in India. These alone are required to complete the statistical history of the mortality, sickness, and diseases affecting white troops within the tropical zones at one and the same period.

*Epilepsy: its Symptoms, Treatment, and Relation to other Chronic Convulsive Diseases.* By J. RUSSELL REYNOLDS, M.D., London, Assistant-Physician to University College Hospital. 8vo. London, 1861. Pp. 360.

The work of Dr. Russell Reynolds upon Epilepsy and its Relation to other Chronic Convulsive Diseases, is of such merit as at once to entitle its author to a foremost place among the most distinguished of the advanced school of modern medicine. Following upon Dr. Radcliffe's celebrated lectures at the College of Physicians, the practical work of Dr. Sieveking, the experimental researches of Kussmaul and Tänner, of Bernard, and of Brown-Séquard, Dr. Reynolds' work, while not superseding other treatises, is excellent as an account of the author's own experience, well written, and well ordered. He is not so original as Dr. Radcliffe, nor so fertile in expedient and research as the French physiologists, but he is thoroughly practical and safe. Indeed, this work is just such an one as might have been predicated from his education and his experience, his antecedents and his industry. In common with most men of the present school, he is addicted to the numerical method.

By means of it he labours to show that some general principles enunciated and received upon epilepsy are subject to exceptions sufficient to invalidate them. Further, he employs statistics to adduce the validity of principles antagonistic to those currently received. In the discussion of the phenomena peculiar to epilepsy, he scrupulously eliminates all attendant signs and symptoms which, held, for example, by the late Dr. Todd, to sanction the admission of certain spasmodic seizures into the epileptic group, are but simulations of epilepsy proper; and, furnished with eighty-eight well-marked cases of the real disease, Dr. Reynolds proceeds to a rigid investigation of their etiology, their special character, their pathological and general features. By this method of evaluating his results, he finally attempts to bring the disease of which he treats within the scope of exact knowledge.

Considering the extended nature of the subject of convulsions, according as their apparent causes are investigated, it is satisfactory to find in this work a clear and definite view of the immediate nature of such deviations from healthy muscular action. It is no small proof of an author's physiological foresight that he can discard all irrelevant and cumbersome correlative conditions, and take his stand on an apparent aphorism. Is healthy muscular function inherent or derived? Is contractility itself an integral property of muscular tissue, or imparted by nerve-association? Dr. Reynolds does not hesitate to express his belief that, whatever the subjective features of convulsions may be, their prominent or immediate cause is an abnormal increase in the nutritive changes of the nerve centres. He arrives at this hypothesis by observations upon muscular innervation and contraction, correlated, as these he believes are, to assimilation and disintegration. Excess of the latter functions brings with it exaggerated manifestations of the former; and the mutual dependence of these several vital conditions, he believes, may be positively estimated in like manner as the intensity and amount of a voltaic current may be calculated from the quantity of metal consumed at the poles, and the intensity of the chemical action. If the nutritive processes are accelerated or increased, there is a corresponding augmentation of the vital result. "In the former case the wire may become heated; in the latter, instead of simple tonicity, there is spasm."

On the one side, therefore, according to Dr. Reynolds, is spasm, clonic or tonic; on the other, increase in nutritive change. So that the author begs the question of the dependence of muscular contraction upon nerve influence. Most probably such contraction has such dependence. But the views of Dr. Radcliffe are not so unsupported by evidence or so illogically reasoned out as not to render it incumbent on an author on this special subject to show that the function of nerve is not of a strictly limiting and controlling character. And apart from this, assimilation and disintegration, coupled in so intimate and equational a relationship, are *not* always mutually correlated. Assimilation may occur independently of disintegration. Disintegration often *does* occur independently of assimilation. And we cannot help observing that the last stage of uræmia, and other blood-poisonings, in which there is certainly no increase in nutritive change, but a simple waste of tissue and perversion of natural chemical conditions, is very frequently not only accompanied by convulsions, but evidenced by them. The augmentation, therefore, in degree of nutritive change, whose vital effects are secretion, motion, and functional activity, can scarcely, we believe, suffice to account for spasm.

The remote causes of convulsion are in Dr. Reynolds' opinion, consequently such as to induce this abnormal increase in nutritive change. Kussmaul and Tänner have, however, clearly shown that compression of the carotids will bring about epilepsy; and Brown-Séquard has also shown that certain sections of the cord will induce directly spasm with unconsciousness.

The experiments of Claude Bernard with respect to this object may perhaps—inasmuch as they are directed more to the effects of irritative lesion—be held to confirm the view of Dr. Reynolds, since irritation may not unreasonably be supposed to be accompanied by nutritive changes; but the results of the former experimentalist show that when nutritive change is diminished and nearly annihilated, spasm and convulsions are brought about; and in fact the less the change the stronger we may expect convulsions to be. The organ affected in epilepsy is, according to our author, the medulla oblongata on the upper part of the spinal axis. The evidence of this he decides from the attacks of the mitigated seizure, and from the abortive seizures without loss of consciousness, which point out the medulla as the most frequent starting-place of spasmodic action. Is loss of consciousness essential to the epileptic seizure? If so, we should ourselves refer the seat of the disease as much to the hemisphere as to the medulla. The disturbances of respiration, seldom unobserved, show, we agree with our author, that the medulla is as a rule implicated. Is the morbid change one of structure? Scarcely, or there could be no complete remission. The disturbance is functional. The essential elements of a convulsion are present in health, and the unconsciousness occurs in sleep. The two combined, and in an aggravated form, may reasonably therefore be held to indicate an exaggeration of natural conditions; but a deeper inquiry should elicit whether those conditions resulting in a seizure are present during an intermission. This should be the aim of all pathologists. What the results are we see. What the causes of these results are we mostly may trace. What the locus on which these expend themselves we fairly may surmise. But we are in absolute darkness as to the amount of the masked persistence of the inducing causes and subdued abnormal deviation from the healthy state which abide, dormant and recruiting, during comparative health. The chapter on the symptoms of epilepsy is complete in a theoretical point of view; from a practical point of view almost redundant. Inasmuch as, which we have already observed, Dr. Reynolds treats but of epilepsy proper—the chronic disease characterized by the occasional and temporary existence of loss of consciousness with or without evident muscular contraction—he is at great pains to describe at length the differential diagnosis. He establishes practical distinctions between epilepsy, simulated convulsions, syncope, hysteria, catalepsy, eccentric convulsions, diathetic convulsions, urinæmia, chronic alcoholism, and other poisoned states of the system, and such seizures induced by diseases of the nervous centres and their coverings, whether brought about by softenings, inflammations, hypertrophies, or foreign bodies.

Dr. Reynolds has found opium, conium, belladonna, henbane, and stramonium both to delay the attacks and diminish their severity. He speaks highly of the Indian hemp, disparagingly of cotyledon umbilicus. Of castorem and turpentine, so commended by Dr. Radciffe, his experience has been limited, and chloroform he believes to delay the attack for a time, but to exert no permanently good influence.

*Transactions of the Epidemiological Society of London.* Vol. I., Part 2. 8vo. London, 1862. Pp. 129—256.

The second part of these Transactions fully bears out the impression we had formed, on the publication of the first part, of the great advantage to the profession of printing and publishing the transactions of the society as a separate work. In addition to the address of the president at the opening of the eleventh session, and which includes Dr. M'William's valuable report on the epidemics of the preceding year, the list of papers contained in the present number is most varied and interesting. (1.) Professor Simpson, of Edinburgh, favours us with certain "Notices of the Appearance of Syphilis in Scotland, in the last years of the 15th century." This elaborate paper abounds with matter full of the most curious interest, and throws additional light upon disputed points in the etiology of the disease at the time to which the paper refers. (2.) Dr. Hopffus, Surgeon of the Second Class on the Staff of the Cape de Verd Sanitary Department, communicates certain brief "Notes on the Epidemic of Cholera Morbus at the Island of St. Jago, Cape de Verd, in 1856." (3.) Dr. Milroy discusses the important question of "The Influence of Contagion on the Rise and Spread of Epidemic Diseases." This is a graphic and thoughtful summary of the subject, which cannot be too widely known. (4.) Dr. Bryson, Inspector-General of Hospitals and Fleet, tells the story of "The Recent Introduction of Yellow Fever into Port-Royal, Jamaica." This highly important paper is based upon reports and letters received from the inspectors of the *Icarus*, the *Imaum*, and *Barracouta*, and from the Deputy Inspector-General of the Naval Hospital at Port-Royal. The author concludes, from evidence which he thinks cannot well be disputed, that (1) the crew of the *Icarus* brought the fever to Port-Royal; (2), that they infected the boat's crew sent from the *Imaum* to remove the sick to the hospital; (3), that the boat's crew next infected their shipmates in the *Imaum;* (4), in the same manner the sick of the *Icarus* communicated the fever to the boat's crew sent from the *Hydra*, and that they subsequently infected their shipmates; (5), that the crew of the *Barracouta* were infected by the supernumerary men and boys sent from the *Imaum;* and (6), that the yellow fever patients sent to the hospital infected other patients who had been in the hospital previously to the arrival of the *Icarus*. (5.) "Further Observations on Scarlet Fever," by Dr. D. W. Richardson, refer (*a*) to the reason why seasons influence the spread of scarlet fever; (*b*), to the cause of the differences of types of the disease; (*c*), the connexion of scarlet fever with acute rheumatic fever; (*d*), the chemical pathology of scarlet fever; and (*e*) the poison of scarlet fever in relation to its propagation and mode of action. (6.) "Observations on the Climatology, Topography, and Diseases of Hong Kong, and the Canton River Station," by Dr. W. R. E. Smart, Deputy Inspector-General of the Royal Naval Hospital, Bermuda, is a valuable and most laborious monograph, abounding with important facts. (7.) "On Fever in the Zambesi," a note from Dr. Livingstone to Dr. M'William, on the benefits derived from the use of a combination of jalap, rhubarb, calomel, and quinine in the treatment of fever in his African journeys, is followed by a highly interesting summary, by Dr. M'William, of the mortality occurring in several of the more important African expeditions; for example, that to the Congo in 1816; to the Zambesi, from Captain Owen's ship, in 1829; to the Niger, under Laird, Lander, and Oldfield, in 1832; and to the Niger, under Captain Trotter, in 1845. Dr. M'William also gives a summary of the mortality in the *Eclair* in 1845, and the dreadful loss sustained by two detachments of soldiery at Gambia in 1825; as also that suffered by Mungo Park's party in the last journey of that great traveller into the interior of Africa. (8.) "On Yellow Fever in the West Indies and West Coast of America," by Dr. Archibald Smith. (9.) "On the Recent Introduction of Fever into Liverpool by the Crew of an Egyptian Frigate," by W. H. Duncan, M.D., Medical Officer of Health to the Corporation of Liverpool. The facts contained in this most important paper were reviewed at length by Dr. Milroy, in the preceding volume of this Journal, p. 552.

It will be seen, then, that the contents of this number of the Transactions of the Epidemiological Society are of unusual interest. We observe that the society, contrary to the course it adopted with the first number, has permitted this to be published and sold in the ordinary course of trade. The society has done well to adopt this course, and place its Transactions within reach of every one; and we trust that the profession will show a generous appreciation of the work thus placed within its reach.

*The Breath of Life, or Mal-Respiration and its Effects upon the Enjoyments and Life of Man.* (Manu-graph.) By GEORGE CATLIN, Author of "Notes of Travels amongst the North American Indians," Small 8vo. London, 1861. Pp. 75.

If any of our readers are tinctured with a fancy for curious books; if any can thoroughly enjoy exceptional physiological speculation; if any can rightly appreciate the fun which may be dashed off in pen-and-ink sketches, let them hasten to their bookseller and secure Mr. Catlin's manu-graph without delay. He, scorning vulgar type, has had his own manuscript, written with loving care, and illustrated with many vigorous and amusing sketches, transferred to the impressible stone, and so printed off for our delectation and instruction. This little work is, in short, lithographed, and we should think, of its kind, pretty nigh unique. Mr. Catlin writes in the best of faith, and it is impossible to read his essay without entertaining much kindly respect for the author, whatever may be thought of his theory.

Mr. Catlin, having been especially struck with the fact, of which he had been assured by breeders, that the horse, the dog, the ox, and other animals were little subject to fatal diseases of the respiratory and digestive organs, diseases of the spine, idiocy, deafness, or decay of the teeth; having also noticed during his extensive travels among, and long association with, the native races of North America, who still retained their primitive condition, a like immunity from these diseases, and especially an absence of that premature mortality which is so sadly manifested among the young in large towns, he set himself to work to discover the cause or causes of these striking peculiarities. The results of his researches are now before us.

The great cause of this notable difference between the health—the vital resistance of the savage and the brute and the civilized man, arises, he believes, from "the simple neglect to secure the vital and intended advantages to be derived from quiet and natural sleep—the great physician and restorer of mankind, both Savage and Civil, as well as of the brute creation." Now this, Mr. Catlin asserts, is engendered by the habit acquired in civilized life, from carelessness in infancy, of *sleeping with the mouth open.* This is the true secret of several of the deadliest ills to which man is exposed. This it is which leads to that deterioration of the constitution, of which the lung-affections which exercise so fatal an influence over young children, spinal diseases, diseases of the digestive organs, idiocy, and deafness, are the sad indications. If our readers ask in what manner these deadly results can be brought about by a cause apparently so simple, let them read:—

"We are told that 'the breath of life was breathed into man's nostrils';—then why should he not continue to live by breathing it in the same manner?

"The mouth of man, as well as that of the brutes, was made for the reception and mastication of food for the stomach, and other purposes; but the nostrils, with their delicate and fibrous linings, for purifying and warming the air in its passage, have been mysteriously constructed, and designed to stand guard over the lungs—to measure the air and equalize its draughts, during the hours of repose.

"The atmosphere is nowhere pure enough for man's breathing until it has passed this mysterious refining process; and therefore the imprudence and danger of admitting it in an unnatural way, in double quantities, upon the lungs, and charged with the surrounding epidemic or contagious infections of the moment.

"The impurities of the air, which are arrested by the intricate organizations and mucus in the nose, are thrown out again from its interior barriers by the returning breath; and the tingling excitements of the few which pass them, cause the muscular involitions of sneezing, by which they are violently and successfully resisted.

"The air which enters the lungs is as different from that which enters the nostrils as distilled water is different from the water in an ordinary cistern or a frog-pond. The arresting and purifying process of the nose upon the atmosphere, with its poisonous ingredients passing through it, though less perceptible, is not less distinct, nor less important than that of the mouth, which stops cherry-stones and fish-bones from entering the stomach. . . . (P. 23.)

"Against the approach of these things [poisonous matters and animalculæ in the air] to the lungs and to the eye, Nature has prepared the guard by the mucous and organic arrangements, calculated to arrest their progress. Were it not for the liquid in the eye, arresting, neutralizing, and carrying out, the particles of dust communicated through the atmosphere, man would soon become blind; and, but for the mucus in his nostrils absorbing and carrying off the poisonous particles and effluvia, for the protection of the lungs and the brain, mental derangement, consumption of the lungs, and death would ensue.

"How easy, and how reasonable, it is to suppose then that the inhalation of such things to the lungs, through the expanded mouth and throat, may be a cause of consumption, and the other fatal diseases attaching to the respiratory organs; and how fair a supposition, also, that the deaths from the dreadful epidemics, such as cholera, yellow fever, and other pestilences, are caused by the inhalation of animalculæ in the imperfect districts; and that the victims to those diseases are those portions of society who inhale the greatest quantities of those poisonous insects to the lungs and the stomach.

"In man's waking hours, when his limbs, and muscles, and his mind, are all in action there may be but little harm in inhaling, through the mouth, if he is in a healthy atmosphere, and at moments of violent action and excitement, it may be necessary. But when he lies down at night to rest from the fatigues of the day, and yields his system and all his energies to the repose of sleep, and his volition and all his powers of resistance are giving way to its quieting influence, if he gradually opens his mouth to its widest strain, he lets the enemy in that chills his lungs—that racks his brain—that paralyses his stomach —that gives him the nightmare—brings him imps and fairies, that dance before him during the night; and during the following day, headache, toothache, rheumatism, dyspepsia, and the gout." . . . . (P. 25.)

"All persons going to sleep should think—not of their business, not of their riches or poverty, their pains or pleasures—but of what are of infinitely greater importance to them, their lungs, their best friends, that have kept them alive through the day, and from whose quiet and peaceful repose they are to look for happiness and strength during the toils of the following day. They should first recollect that their natural food is fresh air; and next, that the channels prepared for the supply of that food are the nostrils, which are supplied with the means of purifying the food for the lungs, as the mouth is constructed to select and masticate the food for the stomach. The lungs should be put to rest as a fond mother lulls her infant to sleep; they should be supplied with vital air, and protected in the natural use of it; and for such care, each successive day would repay in increased pleasures and enjoyments.

"The lungs and the stomach are too near neighbours not to be materially affected by abuses offered to the one or the other; they both have their natural food, and the natural and appropriate means prepared by which it is to be received. Air is the especial food of the lungs, and not of the stomach. He who sleeps with his mouth open draws cold air and its impurities into the stomach as well as into the lungs, and various diseases of the stomach, with indigestion and dyspepsia are the consequences. Bread may almost as well be taken into the lungs, as cold air into the stomach.

"A very great proportion of human diseases are attributed to the stomach, and are there met and treated; yet I believe they have a higher origin, the lungs; upon the regular and healthy action of which, the digestive as well as the respiratory and nervous systems depend: the moving, active principle of life, and *life itself*, are there; and whatever deranges the natural action at that fountain affects every function of the body." (P. 27.)

The Red Indian, from a baby, taught by the mother with anxious care, sleeps with his mouth shut; so does the brute. Let us do likewise, and Mr. Catlin predicts that the "next generation would be a re-*generation* of the human race." (p. 73.) Would that it might be so; but not questioning Mr. Catlin's facts about the Red Indian's habits, we cannot admit his assumptions about brutes—at least about cattle. Is not Mr. Catlin aware of the frightful ravages which have of late years been committed among cattle in this country and throughout Europe, by the too familiar "lung-disease"—epizootic pleuropneumonia? Is it possible, then, that the domesticated cattle of civilized life have acquired the same bad habit of sleeping with the mouth open as civilized men and women?

Let not, however, our outline of Mr. Catlin's theory deter our readers from making a personal acquaintance with his book. If it should have this result, it will be to their loss. "No person on earth," says Mr. Catlin in his diminutive preface of four lines, "who reads this little work will condemn it: it is only a question how many millions may look through it and benefit themselves by adopting its precepts."

*On the Health of Merchant Seamen.* By J. O. McWilliam, M.D., F.R.C.P., C.B., R.N., Medical Inspector, Her Majesty's Customs. London, 1862. pp. 10.

This was a paper read before the last meeting of the Social Science Association, and in which Dr. McWilliam broke entirely new ground. Our knowledge of the state of health of the seamen employed in the mercantile marine was previously of the vaguest and most unsatisfactory character. Dr. McWilliam has now, to some extent, raised the veil of difficulties which obscured the question, and brought to light a state of things which calls for a fuller inquiry than is within the power of any one individual to carry out unaided, however generous of his labour. The statistics of mortality in the mercantile marine, collected by Dr. McWilliam, although of necessity yielding a ratio of deaths considerably below the real amount, show an annual average, for foreign voyages, all but equal to that of the Royal Navy (notwithstanding that merchant seamen escape the evils peculiar to the latter service, arising from long exposure to tropical climates); and for the home voyages much in excess.

Surely this is a question which ought to secure the active co-operation of the different chambers of commerce in the kingdom for its solution, seeing how nearly their interests are affected by it.

*An Effectual and Simple Remedy for Scarlet Fever and Measles: with an Appendix of Cases.* By Charles Witt, M.R.C.S. 3rd Edition. London, 1862.

Dr. Witt's practical experience of the great, almost specific, value of sesquicarbonate of ammonia in the treatment of scarlet fever, derives increased interest from the more recent researches and observations of Dr. B. W. Richardson.

*Practical Medical Precepts for Plain People.* By ALFRED FLEISCHMANN, Fellow of the Obstetrical Society, and late Physician-Accoucheur Assistant to King's College Hospital. (Davies.)

This is a broadsheet of simple, but all-important medical precepts adapted for the walls of cottages, and which would prove of the greatest assistance to clergymen and lady-visitors in carrying out their charitable duties.

*In Memoriam:* 1858, 1859, 1860, 1861.

A reprint, from the pages of the last number of the *Dublin Quarterly Journal of Medicine*, of the elegantly written article by Dr. Stavely King, on Sir Philip Crampton, Robert Harrison, Dr. F. H. Montgomery, Sir Henry Marsh, William H. Porter, James W. Cusack, and Francis Rynd.

Take them, O Death! and bear away
  Whatever thou canst call thine own!
Thine image, stamped upon this clay,
  Doth give thee that, but that alone!

Take them, O Grave! and let them lie
  Folded upon thy narrow shelves
As garments by the soul laid by,
  And precious only to ourselves!

